# JAK Inhibition Prevents Bone Loss and Reduces Inflammation in Experimental Periodontitis

**DOI:** 10.1111/jre.70042

**Published:** 2025-10-03

**Authors:** Mariely A. Godoi, Fábio R. M. Leite, Angelo C. Camilli, Karen G. A. Gonzales, Vitória B. Costa, Iolanda A. F. de Matos, Evangelos Papathanasiou, Morgana R. Guimarães‐Stabili

**Affiliations:** ^1^ Department of Diagnosis and Surgery, School of Dentistry at Araraquara Sao Paulo State University (UNESP) Araraquara Brazil; ^2^ Oral Health Academic Clinical Programme Duke‐NUS Medical School Singapore Singapore; ^3^ National Dental Research Institute Singapore National Dental Centre Singapore Singapore Singapore; ^4^ Department of Periodontology Tufts University School of Dental Medicine Boston Massachusetts USA

**Keywords:** bone resorption, cytokines, immunomodulation, Janus kinase inhibitors, periodontitis, signal transduction

## Abstract

**Aims:**

This study aimed to investigate the role of Janus kinase (JAK) signaling in the pathogenesis of periodontitis by evaluating the effects of pharmacological inhibition of JAK isoforms (JAK1 and JAK3) on periodontal inflammation and ligature‐induced alveolar bone loss.

**Methods:**

Experimental periodontitis (EP) was induced by ligature placement around the mandibular first molars of rats. Concurrently, animals (*n* = 10 per group) received either a JAK1‐3 inhibitor (JAK1‐3i group), a JAK3 inhibitor (JAK3i group), or distilled water (EP group) via daily intragastric gavage for 7 days. A control group received only distilled water without ligature placement. Following euthanasia, the mandibles were evaluated using microcomputed tomography for bone loss, stereometric analysis for inflammatory infiltrate and blood vessels, Second Harmonic Generation Microscopy for collagen quantification, and immunohistochemistry to quantify CD45+ and CD3+ cell populations. Gingival tissues were assessed for inflammatory markers by RT‐qPCR (*Il‐6*, *Tnf‐α*, and *Rankl*) and ELISA (TNF‐α).

**Results:**

Ligature placement resulted in significant alveolar bone loss, increased osteoclast numbers, leukocyte infiltration, extracellular matrix degradation, and elevated expression of inflammatory markers. Treatment with both JAK1‐3i and JAK3i effectively prevented bone loss and reduced osteoclast numbers. Histological and stereometric analyses showed reduced inflammatory infiltrate and improved tissue organization in both treated groups. JAK1‐3i preserved collagen content more effectively and significantly reduced the number of CD45+ cells. Compared to the Experimental Periodontitis (EP) group, both inhibitors significantly downregulated the mRNA expression of *Il‐6*, *Tnf‐α*, and *Rankl*, and also reduced TNF‐α protein levels in gingival tissues.

**Conclusion:**

Collectively, the findings establish a mechanistic link between JAK signaling and inflammation‐driven periodontal tissue destruction, providing new insights into the cellular and molecular events underlying the pathogenesis of experimental periodontitis.

## Graphical abstract

The pharmacological inhibition of JAK prevented alveolar bone loss and protected periodontal tissues by limiting inflammatory cell infiltration, preserving collagen structure, and maintaining low levels of pro‐inflammatory mediators. These findings highlight the therapeutic potential of targeting JAK/STAT signaling to modulate periodontal inflammation and prevent tissue destruction.

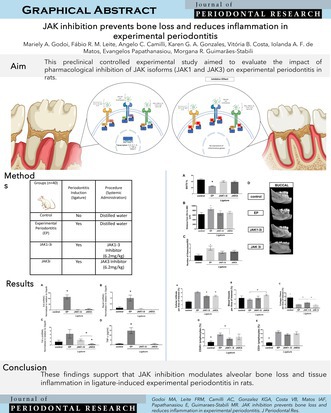

**Summary**

- Background
  ○ The JAK pathway plays a pivotal role in regulating cytokine production, which is critical in the development of inflammatory diseases. Periodontitis, an inflammatory disease leading to bone loss and tissue destruction, involves complex immune responses.
- Added value of this study
  ○ This study explored the effects of JAK inhibitors on experimental periodontitis in rats and demonstrated that JAK inhibitors (JAK1‐3i and JAK3i) significantly reduced inflammatory markers, prevented alveolar bone loss, and enhanced tissue repair in periodontitis. Notably, JAK inhibition decreased cellular infiltration, enhanced collagen matrix formation, and increased blood vessel density in periodontal tissues, showing the potential for targeting the JAK pathway in inflammatory conditions like periodontitis.
- Clinical implications
  ○ These findings provide mechanistic evidence that JAK signaling contributes to periodontal inflammation and bone loss. By elucidating the biological role of this pathway, the study supports future investigation of JAK inhibitors as potential adjunctive therapies in periodontitis.

## 1 Introduction

Periodontitis is a chronic inflammatory disease characterized by the progressive destruction of the tooth‐supporting tissues, triggered by a dysbiotic microbial biofilm and modulated by host immune and inflammatory responses [1]. Microorganisms release virulence factors, such as lipopolysaccharides (LPS) and antigens, which activate the host immune response through cytokines, chemokines, and transcription factors. While the immune response has a primarily protective role, it is also responsible for most of the tissue degradation observed in periodontitis [2, 3].

Current periodontal treatment focuses mostly on the removal of biofilm, but this approach often fails to achieve satisfactory and stable clinical outcomes, particularly in those with chronic non‐self‐resolving inflammatory periodontal lesions. In these lesions, neutrophils resist clearance, prompting a shift in macrophages towards a proinflammatory phenotype and activating the adaptive immune response, which leads to lymphocyte accumulation [4]. Complementary therapies that modulate the immune response and trigger the resolution of these chronic lesions have been explored as potential adjunct strategies to achieve better long‐term results [5].

Modulating components of the host immune response can help restore the balance between pro‐ and anti‐inflammatory mediators, arresting periodontitis progression and promoting an environment conducive to inflammation resolution [1]. Additionally, controlling the inflammatory process limits the availability of nutrients to the microbiota, favoring the reversal of dysbiosis, and stimulating the recovery of a microbial community compatible with periodontal health [1].

Agents such as bisphosphonates, omega‐3 supplements, polyphenols like curcumin and resveratrol, sub‐antimicrobial doses of doxycycline and nonsteroidal anti‐inflammatory drugs have demonstrated promise when combined with non‐surgical periodontal therapy, reducing disease progression and promoting inflammation resolution [2].

Considering the role of cytokines in inflammatory diseases [3, 6, 7], immunomodulatory therapies targeting the inhibition of specific cytokines have shown success in treating conditions such as rheumatoid arthritis, cancer, and osteoporosis. Similarly, these therapies have demonstrated efficacy in animal studies using experimental models of periodontitis [8, 9]. An alternative approach for controlling the overexpression of pro‐inflammatory mediators involves regulating signaling pathways responsible for their expression. This strategy is advantageous because the expression of multiple inflammatory cytokines often depends on the activation of a few signaling pathways. In other words, regulating a single pathway can impact the expression of several cytokines. Additionally, the activation of signaling pathways is typically rapid and transient, allowing the regulation of one pathway with minimal adverse effects on normal cellular processes [9].

The Janus kinase (JAK) family comprises four tyrosine kinases (JAK1, JAK2, JAK3, and TYK2) that play a central role in cytokine receptor signaling and immune regulation [10]. These kinases associate with the cytoplasmic domains of transmembrane receptors and, upon activation by cytokines, initiate phosphorylation cascades that ultimately regulate the expression of genes involved in inflammatory responses including those relevant to periodontitis [11, 12].

Given the relevance of the JAK signaling in modulating inflammatory cytokines, pharmacological JAK inhibitors have been approved by the Food and Drug Administration (FDA) for the treatment of inflammatory diseases such as rheumatoid arthritis, multiple sclerosis, and inflammatory bowel diseases [11]. These small molecules block cytokine signaling by interfering with JAK enzymatic activity and offer advantages over monoclonal antibodies such as oral administration, short plasma half‐life, reduced immunogenicity and linear pharmacokinetics [11]. They represent a promising alternative to conventional immunomodulators, which often have substantial recurrence rates [11, 12].

Based on promising results of JAK inhibitors in clinical and pre‐clinical studies of inflammatory osteolytic diseases, a few studies have evaluated their therapeutic potential in preventing periodontitis [13, 14]. Although results indicate a relevant role for JAK in periodontitis pathogenesis, the data remain limited and inconsistent. Therefore, the goal of this study was to investigate the therapeutic effects of JAK inhibitors on ligature‐induced periodontitis in rats, focusing on the prevention of periodontal tissue degradation.

## 2 Methods

Experimental protocols were approved by the Ethics Committee for Animal Use (CEUA—process 27/2021) of the School of Dentistry at Araraquara—UNESP and were conducted in accordance with the guidelines of the Brazilian College for Animal Experimentation (COBEA). The study adheres to the ARRIVE (Animal Research: Reporting of In Vivo Experiments) guidelines.

### 2.1 Animals

Forty male rats ( *Rattus norvegicus* albinus, Holtzman), aged 7–8 weeks and weighing between 100 and 200 g were utilized in this study. The animals were housed in polypropylene cages (5 animals/cage) in a conventional animal facility, maintained at 21°C ± 1°C, with 65%–70% humidity, under a 12‐h light/dark cycle. The animals received pelleted food (Labina, Purina, São Paulo, Brazil) and water ad libitum.

### 2.2 Induction of Experimental Periodontitis

Experimental periodontitis was induced by placing ligatures bilaterally around the mandibular first molars, as previously described [15]. Animals were sedated using an intravenous injection of 0.08 mL ketamine [Paulinia, Sao Paulo, Brazil] and 0.04 mL xylazine hydrochloride [Tambore, Sao Paulo, Brazil] per 100 g body weight. The ligatures (cotton thread no. 24, 1 mm thick) were positioned while the animals were on an operating table and remained in place for the 7‐day experimental period till euthanasia. This timeline was selected based on prior studies showing significant inflammation and bone resorption within 7 days of ligature placement [16, 17, 18]. All animals were euthanized using an overdose of ketamine and xylazine under general inhalation anesthesia.

### 2.3 Effect of JAK Inhibition in Ligature‐Induced Periodontitis

To investigate the effect of JAK inhibition on the development of experimental periodontitis, animals were randomly assigned to four groups (*n* = 10 per group), as described in Table S1: control (no ligature, distilled water ingestion by gavage), experimental periodontitis (EP) (ligature, distilled water ingestion), JAK1‐3i (ligature and JAK1‐3 inhibitor ingestion) and JAK3i (ligature and JAK3 inhibitor ingestion). The impact of JAK inhibition was evaluated after a 7‐day experimental period by comparing alveolar bone loss and inflammatory changes among healthy control, untreated periodontitis, and JAK inhibitor‐treated groups. Sample size calculation was based on previous data, where bone volume was the primary outcome, measured by bone microtomography in rats [15]. A minimum sample size of eight animals per group was established to ensure 80% statistical power. To account for potential losses during the experimental period, we opted to use a sample size of 10 animals per group. The calculations were based on an alpha level (*α*) of 0.05, a standard deviation of 2.5%, and an equivalence limit (Δ) of 4% [15]. With 10 animals per group, the study achieved a power of 90%.

Two types of JAK inhibitors were administered: a commercially available selective inhibitor for JAK1‐3 isoforms (CP‐690‐550—Tofacitinib; Cayman Chemical, MI, USA), and a JAK3 isoform‐specific inhibitor (TL6‐144). The JAK3 isoform‐selective inhibitor was synthesized and kindly provided by Prof. Nathanael Gray (Stanford University, USA). Prior pharmacological analyses demonstrated the absence of adverse effects with oral administration and effective inhibition of JAK3 in mice [19, 20].

Both inhibitors were diluted in 0.5% carboxymethylcellulose (CMC) and administered by daily intragastric gavage at a dose of 6.2 mg/kg for 7 days, starting on the same day as ligature placement. The dose was determined based on studies using an experimental model of adjuvant‐induced rheumatoid arthritis [21, 22]. Mortality and any physical or behavioral changes were recorded daily. The control group and the EP group received distilled water via the same route (oral gavage), in order to mimic the handling and potential stress associated with the daily administration of inhibitors in the treated groups. A vehicle control group (carboxymethylcellulose 0.5%) was not included, as our prior studies demonstrated no effect of the compound on outcomes similar to those evaluated in this study [15]. Details of the experimental procedures are represented in Figure S1.

### 2.4 Biological Samples

After euthanasia, the lower jaw of each rat was sectioned into two hemi‐mandibles. Left hemi‐mandibles were fixed in 4% paraformaldehyde (Sumaré, Sao Paulo, Brazil) at 4°C for 18 h, transferred to 70% ethanol and stored at 4°C. These samples were used for immunohistochemical and stereometry analysis. Gingival tissue surrounding the first molars on the right side was carefully dissected, frozen at −80°C and later processed for ELISA and mRNA expression analysis. The right hemimandibles were immersed in 70% alcohol and used for microcomputed tomography (μCT) analysis.

### 2.5 Microcomputed Tomography (μCT) Analysis

Right hemimandibles were scanned using a μCT imaging system (Skyscan 1076; Bruker, Kontich, Belgium) using an isotropic spatial resolution of 9 μm, a 0.5 mm aluminum filter, 80 kV voltage, 310 μA current, 195 ms exposure, frame averaging of 3 and a rotation step of 0.4° over 180°. Reconstruction was performed using NRecon software (version 1.6.1.5; Bruker, Skyscan, Belgium), with the following parameters: ring artifact correction equal to 4, 5%‐pixel defect mask, 1% straightening, 26% beam hardening correction and compressed sensor for image conversion at 0.0–0.8. A standardized threshold was applied to distinguish non‐mineralized and mineralized tissues.

A region of interest (ROI) measuring 2.5 mm^3^ was defined in the sagittal plane according to anatomical landmarks: the apex of the distal root of the first molar (apical limit), bottom of the first molar furcation (coronal limit), the most distal aspect of the mesial root of the second molar (posterior limit), and the most mesial aspect of the mesial root of the first molar (anterior limit) (Figure S2). Bone fraction volume (BVF) within this ROI was quantified using CT Analyzer software (version 1.12.4.0; Bruker microCT, Skyscan, Belgium). The first molar furcation region was selected as the ROI based on previous analyses from our research group (unpublished), which demonstrated the greatest differences in BVF values between negative and positive control groups in this region. The ratio of bone volume to total volume (BV/TV) within the ROI was compared across groups by a trained examiner blinded to the experimental conditions. Linear bone loss was measured by calculating the distance between the cementum–enamel junction (CEJ) and the alveolar bone crest (ABC) on the mesial and distal surfaces of the first molars. The analyses were performed by a previously calibrated researcher who was blinded to the experimental groups. The graphs represent the mean values from eight animals per group, combining measurements from both sites.

### 2.6 Histological Processing

The left hemimandibles, containing the first and second lower molars, were decalcified in a 10% EDTA solution (Diadema, Sao Paulo, Brazil) at pH 7.2 for 6–8 weeks. The samples were then embedded in paraffin (Ribeirao Preto, Sao Paulo, Brazil). For the immunohistochemistry analyses, serial sections of 5 μm were mounted on silane‐coated glass slides. For the stereometry and histomorphometric analyses, semi‐serial sections of 4 μm thickness were mounted on standard glass slides and stained with hematoxylin (St Louis, MO, USA) and eosin (Diadema, Sao Paulo, Brazil) (H&E).

### 2.7 Stereometric and Histomorphometric Analysis

Semi‐serial sections with approximately 320 μm intervals were stained with H&E and images were captured under a light microscope (Leica DM 2500, Wetzlar, Germany) at 200× magnification.

For stereometric analysis, an 88‐point grid was superimposed on the connective tissue corresponding area of the histological image between the first and second lower molars. The region of interest was delimited coronally by the most apical portion of the epithelial tissue, apically by the top of the bony crest, and laterally by the most prominent region of the distal root of the first molar. Tissue components, including cellular infiltrate and blood vessels, were quantified as percentages relative to the total number of points counted. Analyses were performed by a blinded examiner, and results were averaged per animal before statistical comparisons across groups.

Histological quantification of osteoclasts was performed on decalcified and paraffin‐embedded sagittal sections of the mandibular first molar region. TRAP (tartrate‐resistant acid phosphatase) staining was conducted using a solution containing sodium acetate, sodium tartrate, and naphthol, followed by incubation at 35°C–40°C for at least 4 h. After washing, the slides were counterstained with Carrazi hematoxylin and mounted. TRAP‐positive multinucleated cells (containing three or more nuclei) in direct contact with the alveolar bone surface were identified and counted as osteoclasts. The analysis was performed in a standardized region of interest, encompassing the furcation area of the first molar and the interproximal region between the first and second molars. Counts were conducted under light microscopy (Leica DM2500) at 200× magnification, by a previously calibrated examiner who was blinded to the experimental groups. The average number of osteoclasts per animal, based on counts in the standardized regions, was calculated and compared between groups.

### 2.8 Second‐Harmonic Generation (SHG) Microscopy

Second‐harmonic generation microscopy was used to quantify the extracellular matrix collagen structure, which undergoes changes during periodontitis progression. Gingival tissue sections stained with H&E were analyzed at the National Institute of Sciences and Technology on Photonics Applied to Cell Biology (INFABiC; State University of Campinas). SHG microscopy was performed using a Zeiss LSM 780‐NLO confocal system (Carl Zeiss AG, Göttingen, Germany), and ZEN 2.1 SP3 software, following the guidelines described by Natal [23]. Quantitative analysis of the percentage of the region of interest, defined according to the stereometric analysis, occupied by collagen in SHG images was performed using ImageJ (http://imagej.nih.gov/ij/). All analyses were conducted by a trained examiner blinded to the experimental groups.

### 2.9 Immunohistochemistry

The presence of an inflammatory process represented by the number of CD45+ leukocytes and CD3+ T lymphocytes was quantified between the first and second lower molars. A biotin‐streptavidin‐HRP‐DAB method (Envision FLEX kit; Dako, Agilent—#K800021‐2, Santa Clara, CA, USA) was used, following the manufacturer's instructions. Three semi‐serial sections obtained in the sagittal plane were analyzed from four animals per experimental group.

Histological sections were deparaffinized in xylene, rehydrated and incubated in 3% hydrogen peroxide to block endogenous peroxidase activity. Antigen retrieval for CD45+ cells (ABclonal #ABclonal # #AB10558—Woburn, MA, USA) was carried out in a pressure cooker using the Target Retrieval solution from the DAKO kit, with high pH for 3 min at 95°C. For CD3+ cells (ABclonal #16669—Woburn, MA, USA), sodium citrate was used in a pressure cooker for 3 min at 95°C, followed by cooling for 15 min. Sections were incubated with primary antibody at 1:200 dilution at 4°C in a humidified chamber. For CD45, the primary antibody was incubated for 16 h, while for CD3, the incubation time was 1 h. Primary antibody detection was performed with a biotinylated secondary antibody (N‐Histofine #414191F—Chuo‐ku, Tokyo, Japan) and visualized with DAB chromogen (N‐Histofine #425312F—Chuo‐ku, Tokyo, Japan). Counterstaining was performed with Carrazi's Hematoxylin (St Louis, MO, USA) and coverslips were mounted with Entellan (Sigma‐Aldrich, Burlington, MA, USA).

Images were captured at 200× magnification using a light microscope (Diastar‐Cambridge Instruments—Cambridge Instruments, Buffalo, NY, EUA) under constant brightness, contrast and exposure. Intensity and quantification of labeled cells was performed using the Aperio software (https://www.leicabiosystems.com/digital‐pathology/manage/aperio‐imagescope/) by an examiner blinded to the experimental groups. The same area of interest outlined and described for the stereometric analysis was consistently used for the quantification of immunohistochemical markers.

### 2.10 Real‐Time PCR (qPCR)

Total RNA was extracted from gingival tissues using Trizol (Invitrogen Corp., Carlsbad, CA, USA) according to the manufacturer's instructions. cDNA synthesis was performed using 700 ng of total RNA and a High‐Capacity cDNA Synthesis Kit (Applied Biosystems, Vilnius, Lithuania) with random hexamer primers. The expression of inflammatory genes (*Il‐6*, *Rankl* and *Tnf‐α*—Pleasanton, CA, USA—#433118) was quantified using TaqMan probes and reagents (TaqMan Fast Gene Expression Assays, TaqMan Universal Master Mix, Applied Biosystems, Vilnius, Lithuania) on a StepOne Real‐Time PCR System (Applied Biosystems, Vilnius, Lithuania). All analyses were performed in duplicate, with GAPDH as the endogenous control. Relative gene expression levels were calculated using the comparative CT method.

### 2.11 ELISA Analysis

Total protein was extracted from gingival tissues using T‐PER buffer (Tissue Protein Extraction Reagent, Pierce Biotechnology, Rockford, IL, USA), supplemented with a protease inhibitor cocktail (Protein Stabilizing Cocktail, Santa Cruz, MO, USA). Tissue samples were macerated for 5 min, centrifuged at 13 000 RPM at 4°C, and the supernatant collected. TNF‐α levels were determined using an ELISA kit (R&D Systems—#RTA00, Northeast Minneapolis, MN, USA), following the manufacturer's instructions. Protein concentrations were normalized to total protein content, determined using the Lowry method (DC assay, Bio‐Rad).

### 2.12 Data Analyses

Data were analyzed using GraphPad Prism 6.0 (GraphPad Software Inc., San Diego, CA, USA). The Shapiro–Wilk test was used to confirm the normal distribution of data. One‐way ANOVA followed by Tukey's post hoc test was used for multiple group comparisons. A significance level of *p* < 0.05 was applied to all analyses.

## 3 Results

No animals were lost during the experiment, and there were no significant changes in body weight, or noticeable physical or behavioral alterations indicative of adverse effects associated with the experimental protocol.

### 3.1 JAK Inhibition Prevented Bone Resorption and Reduced the Number of Osteoclasts

**FIGURE 1:**
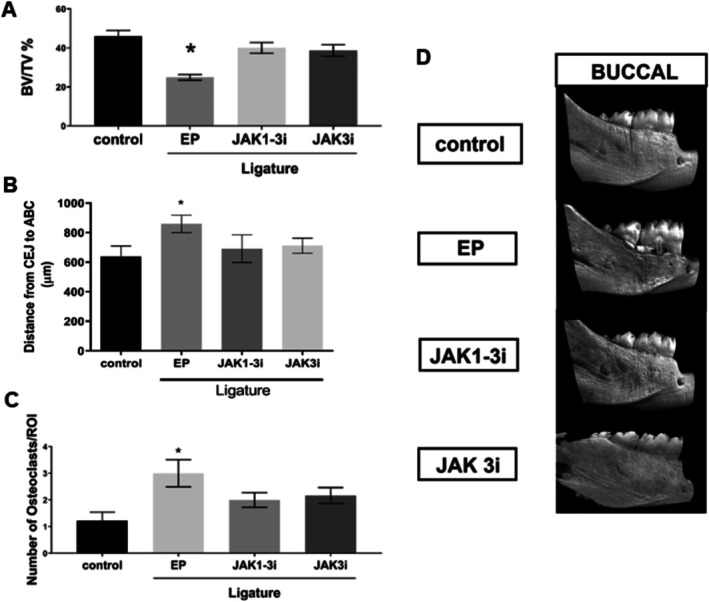
JAK inhibition significantly prevented ligature‐induced alveolar bone loss and reduced osteoclast numbers in rats. (A) Bone volume fraction (BV/TV) was preserved in both JAK1‐3i and JAK3i treated groups compared to the EP group (EP). (B) Linear distance from the cementoenamel junction (CEJ) to the alveolar bone crest (ABC) was significantly increased in the EP group, while treatment with JAK inhibitors prevented this increase. (C) Quantification of osteoclast numbers per animal, based on standardized histological analysis, showed increased osteoclasts in the EP group, which was prevented by JAK inhibition. (D) Representative 3D μCT reconstructions of rat mandibles illustrate bone loss in the EP and bone preservation in treated groups. Data represent findings from 10 animals per group in the μCT analyses and 8 animals per group in the osteoclast quantification. Bars represent mean ± standard error of the mean (SEM). (*) *p* < 0.05 compared to all other groups. ANOVA followed by Tukey's post hoc test.

Three‐dimensional μCT analysis confirmed that ligature placement induced significant alveolar bone resorption in rats. The linear distance between the cementoenamel junction (CEJ) and the alveolar bone crest (ABC) was significantly greater in the EP group compared to the control group (*p* = 0.0031), indicating bone loss. Oral administration of both JAK inhibitors (JAK1‐3i and JAK3i) significantly prevented this linear bone loss compared to the EP group (*p* = 0.0233; *p* = 0.0179, respectively), and the values in the treated groups were comparable to those observed in the control group. Likewise, bone volume fraction (BV/TV) was significantly decreased in the EP group compared to the control (*p* = 0.0004), while this parameter was preserved in both JAK inhibitor‐treated groups, remaining similar to control group levels (*p* = 0.2143 for JAK1‐3i vs. control; *p* = 0.1531 for JAK3i vs. control, Figure 1A,B,D). Experimental periodontitis induction significantly increased osteoclast numbers relative to the control group (*p* = 0.0386). Notably, treatment with both JAK inhibitors effectively prevented this increase (EP vs. JAK1‐3i [*p* = 0.0004]; EP vs. JAK3i [*p* = 0.0026]), maintaining osteoclast numbers at levels not significantly different from those observed in the control group (Figures 1C and Figure S1).

### 3.2 JAK Inhibition Prevented Pro‐Inflammatory Cell Infiltration and Preserved Collagen Structure

H&E‐stained sections demonstrated that ligature placement induced an intense inflammatory reaction, characterized mainly by infiltration of mononuclear and polymorphonuclear leukocytes, particularly beneath the region subjacent to the epithelium of the gingival sulcus and around the alveolar bone crest and furcation region. This was accompanied by connective tissue disorganization and bone fragment formation as a result of the resorption process. In contrast, tissues from animals treated with JAK inhibitors showed reduced cellular infiltration, greater connective tissue organization, increased blood vessel density, and denser collagen fibers. These groups also exhibited better preservation of the bone crest structure. Overall, the histological appearance of JAK inhibitor‐treated groups was more similar to the control group than to the EP group.

**FIGURE 2:**
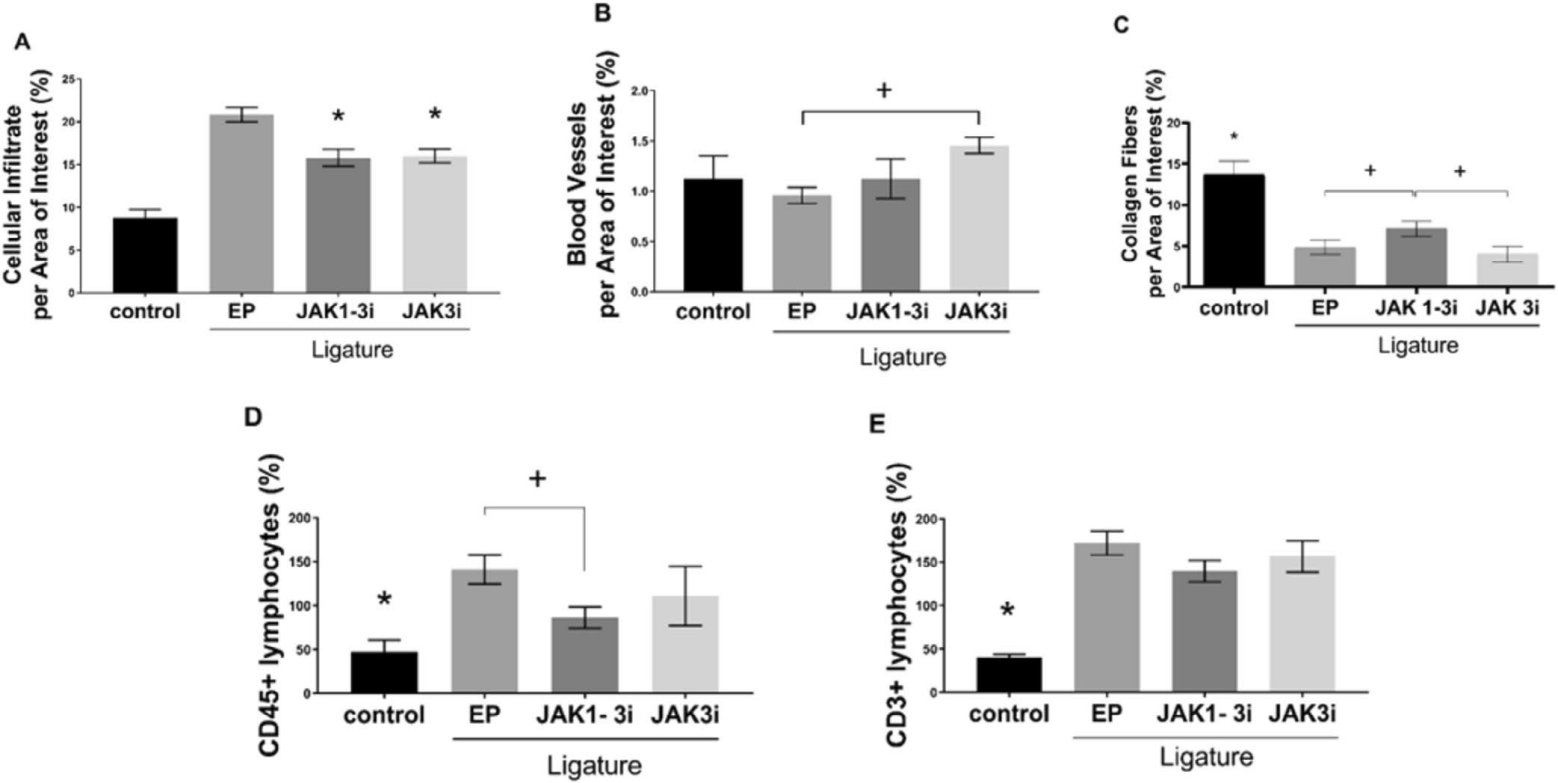
JAK inhibition reduced inflammation in gingival tissues. (A) Stereometric analysis of cellular infiltrate showed that both JAK1‐3i and JAK3i treatments prevented the increase in inflammatory cell infiltration observed in the EP group. (B) The JAK3i group exhibited a significantly higher number of blood vessels compared to the EP group, indicating preservation of vascular structures. (C) Quantitative analysis of collagen content within the region of interest (ROI), assessed by Second Harmonic Generation Microscopy, showed that JAK1‐3i treatment prevented the marked loss of collagen fibers observed in the EP group. (D, E) The percentage of CD45+ and CD3+ cells was evaluated by immunohistochemistry. CD45+ cell levels were significantly lower in the JAK1‐3i group compared to the EP group, indicating protection against leukocyte accumulation. No significant differences in CD3+ cell numbers were observed between treated groups and the EP group. Bars represented mean values, and error bars indicated the standard error of the mean (SEM); (*) *p* < 0.05 vs. all other groups; (+) *p* < 0.05 between the two groups indicated. Data represent analyses of tissue sections from 8 animals per group. Statistical analyses were performed using one‐way ANOVA followed by Tukey's post hoc test.

Stereometric analysis quantitatively supported these observations, showing a significant reduction in cellular infiltrate in both JAK1‐3i‐ and JAK3i‐treated groups compared to the EP (*p* = 0.0097, *p* = 0.128, respectively; Figure 2A). Additionally, animals treated with JAK3i displayed a significant increase in blood vessel density compared to the EP (*p* = 0.0045; Figure 2B), while values in the JAK1‐3i group remained similar to both the control and EP groups. Regarding collagen fibers, all experimental groups exhibited a reduction in collagen content when compared to the control group (control vs. EP; vs. JAK1‐3i; vs. JAK3‐i: *p* < 0.0001; *p* = 0.0004; *p* < 0.0001, respectively), indicating matrix degradation. However, collagen fiber content was significantly better preserved in the JAK1‐3i‐treated group compared to both the EP and the JAK3i group (*p* = 0.0412; *p* = 0.0359, respectively; Figure 2C), although levels did not fully reach those observed in the control group. Notably, JAK3i treatment did not result in a statistically significant improvement in collagen content compared to the EP, suggesting a more limited impact on extracellular matrix preservation.

Immunohistochemical analysis further confirmed the anti‐inflammatory effects of JAK inhibition. The number of CD45+ cells was significantly lower in the JAK1‐3i group compared to the EP group (*p* = 0.0134; Figure 2D), although still elevated relative to the control group. No significant reduction in CD45+ cells was observed in the JAK3i group when compared to the EP. As for CD3+ cells, the control group exhibited significantly fewer T cells than all other groups (control vs. EP, vs. JAK1‐3i, vs. JAK3i: *p* < 0.0001, *p* = 0.0002, *p* = 0.0007, respectively), and no additional differences were found among the experimental groups (Figure 2E). Representative images of these findings are presented in Figure S2. These results are based on analyses of seven animals per group.

### 3.3 JAK Inhibition Reduced the Expression of Inflammatory Markers

**FIGURE 3:**
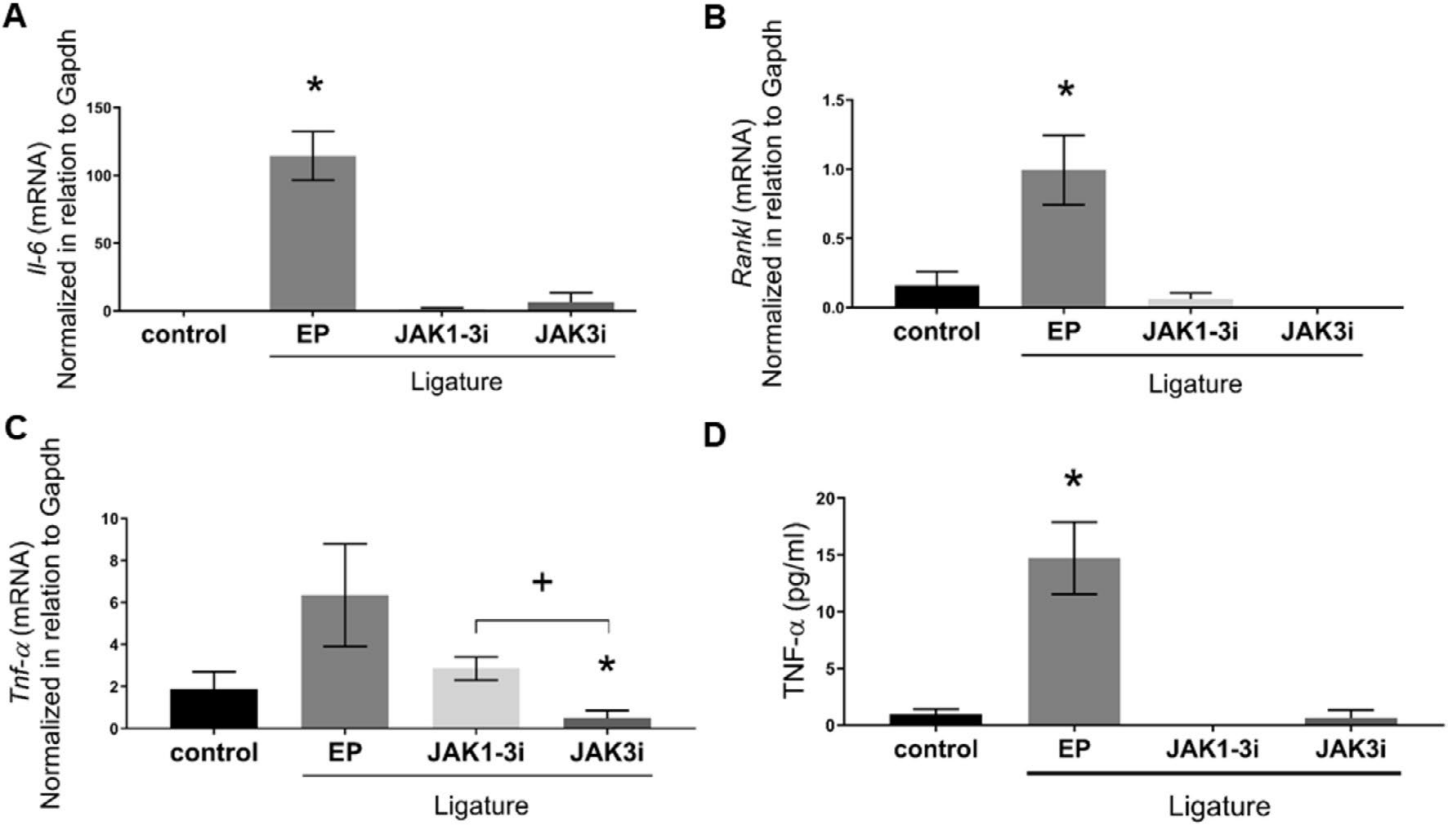
JAK inhibition reduced the expression of inflammatory markers in gingival tissues with experimental periodontitis. (A, B) RT‐qPCR analysis showed a significant reduction in *Il‐6* and *Rankl* gene expression with both JAK inhibitors. Regarding *Tnf‐α* mRNA expression, a significant reduction was observed in the JAK3i group compared to the EP group. Additionally, *Tnf‐α* expression was significantly lower in the JAK3i group than in the JAK1‐3i group (*p* < 0.05). (D) ELISA analysis demonstrated a significant reduction in TNF‐α protein levels with both JAK inhibitors. Bars represented mean values, and error bars indicated the standard error of the mean (SEM); (*) *p* < 0.05 vs. all other groups, (+) *p* < 0.05 between the indicated groups. (#) *p* < 0.05 in relation to the EP group. Data represent analyses of gingival tissue samples from 5 animals per group, evaluated in duplicate. Statistical analyses were performed using one‐way ANOVA followed by Tukey's post hoc test.

Both JAK inhibitors significantly reduced the mRNA expression of *Il‐6* (*p* = 0.0005 for JAK1‐3i; *p* = 0.0065 for JAK3i) and *Rankl* (*p* = 0.0076 for JAK1‐3i; *p* = 0.0045 for JAK3i) compared to the EP group (Figure 3A,B). The expression levels of these markers in the treated groups remained low and were comparable to those observed in the control group, indicating that JAK inhibition preserved baseline inflammatory conditions.

For *Tnf‐α* mRNA, no significant difference was found between the control and EP groups. However, both JAK1‐3i and JAK3i maintained low expression levels of *Tnf‐α*, similar to those in the control group and significantly lower than those in the EP group (*p* = 0.0351, for JAK1‐3i; *p* = 0.0178, for JAK3i; Figure 3C). A significant difference was also observed between the two treatment groups, with JAK1‐3i showing lower expression than JAK3i (*p* = 0.0075).

At the protein level, ELISA analysis revealed a significant increase in TNF‐α in the EP group compared to all other groups (vs. control, vs. JAK1‐3i, vs. JAK3i: *p* = 0.0272, *p* = 0.0182, *p* = 0.0240), confirming an enhanced inflammatory response. Importantly, TNF‐α protein levels remained significantly lower in both JAK inhibitor‐treated groups, similar to the control group (Figure 3D), indicating that JAK inhibition prevented the increase in TNF‐α protein expression induced by experimental periodontitis.

To provide an integrated overview of our findings, a schematic representation summarizing the key molecular pathway and the effects observed upon JAK inhibition in experimental periodontitis is presented in Figure S3. This illustration highlights the effects of JAK inhibition by modulating inflammatory mediators, reducing osteoclastic activity, and preserving tissue integrity.

## 4 Discussion

Systemic administration of JAK1‐3i and JAK3i prevented ligature‐induced bone loss, reduced inflammatory cell infiltration and collagen degradation, and decreased the expression of inflammatory markers in the gingival tissue of rats. These findings align with studies highlighting the relevance of the JAK pathway in regulating multiple cellular processes essential for bone homeostasis and remodeling [24]. Dysregulation of this pathway contributes to excessive bone resorption in conditions such as osteoporosis, rheumatoid arthritis, and periodontitis [25]. Cytokines such as IL‐6, TNF‐α, and RANKL orchestrate osteoclast differentiation and activation within the bone microenvironment, processes deeply influenced by JAK signaling. Downstream targets of JAK activation, including c‐Fos, NFATc1, and TRAP, modulate osteoclastogenesis, while JAK signaling also regulates matrix‐degrading enzymes like cathepsin K and matrix metalloproteinases [26].

Targeting JAK signaling presents a promising therapeutic approach to mitigate bone loss by attenuating excessive osteoclast formation and activity and protecting the connective tissue matrix. In the present study, we demonstrated that pharmacological inhibition of JAK prevented bone resorption, reduced inflammatory cell infiltration, and preserved tissue architecture in an experimental model of periodontitis. Building on prior evidence of the JAK pathway's involvement in various chronic inflammatory and immune‐mediated diseases, our findings further highlight the therapeutic potential of JAK inhibition in this context and may inform research on other conditions where this pathway is central.

It is estimated that non‐surgical periodontal treatment alone fails in approximately 25% of periodontitis cases, resulting in either the arrest or lack of change in progression rates [27]. Therefore, the identification of adjunct therapies that can prevent bone loss in individuals at risk of further periodontitis progression is essential to preserve the dentition and its associated functions such as mastication and speech.

While the role of JAK inhibition in osteolytic diseases has been studied primarily in arthritis models [21, 22], its effects on periodontitis remain less explored. Our results, derived from a study that partly focused on bone resorption during periodontitis induced in rats, align with a previous study conducted on an in vivo model of inflammatory arthritis [22]. This arthritis study utilized JAK1‐3i and observed the prevention of bone resorption through JAK inhibition over the same 7‐day experimental period as ours. This allowed us to evaluate the effects of modulating the pathway during the progression of inflammation [22]. Our findings expand on this understanding, demonstrating that JAK inhibitors reduce bone resorption and the mRNA expression of inflammatory mediators relevant to periodontitis progression, including *Il‐6*, *Rankl*, and *Tnf‐α*, while also reducing osteoclast numbers.

TNF‐α, a key cytokine in inflammatory processes, stimulates fibroblasts to produce matrix metalloproteinases, which degrade the extracellular matrix in both rheumatoid arthritis [28] and periodontitis [29]. TNF‐α and IL‐6 can synergistically collaborate in osteoclastogenesis and enhance osteoclast activity. They act as indirect stimuli for the production of RANKL by other cells, exerting direct effects not only on osteoclast precursor cells but also on mature osteoclasts [30, 31]. The observed reductions in these inflammatory markers with JAK inhibition in our study corroborate findings from other models, linking the inhibition of inflammatory pathways to the preservation of bone and extracellular matrix integrity [32].

In our study, oral administration of JAK inhibitors at a dose of 6.2 mg/kg prevented key pathological changes associated with periodontitis, as evidenced by the preservation of collagen content, reduction of inflammatory infiltrate, and maintenance of vascular density in gingival tissues. This maintained vascularization may favor a faster resolution of inflammation by ensuring adequate oxygen and nutrient supply, thereby contributing to tissue homeostasis [33]. These anti‐inflammatory effects align with findings from studies using JAK1–3 inhibitors (e.g., tofacitinib) in chronic inflammatory conditions such as ulcerative colitis and psoriasis, where JAK inhibition reduced inflammation [13, 34, 35, 36].

Consistent with these findings, analysis of cellular profiles in treated animals revealed a marked reduction in total leukocyte infiltration (CD45+) and a significant decrease in osteoclast numbers, while CD3+ T lymphocyte levels remained unchanged. This pattern observed during the 7‐day experimental period suggests that, under these specific conditions, JAK inhibition primarily affected innate immune cells, such as neutrophils and macrophages, which are key mediators of early periodontal inflammation. In contrast, adaptive immune cells like T lymphocytes appeared less responsive within this timeframe. Additionally, we directly quantified osteoclast numbers and observed a significant reduction following JAK inhibition, supporting a direct effect on bone‐resorbing cells. Although other resident periodontal cells, including fibroblasts and endothelial cells, were not specifically evaluated in this study, these stromal cell types express components of the JAK pathway and may also be responsive to its inhibition. Their potential modulation could contribute to the observed preservation of tissue architecture and attenuation of local inflammation. Overall, these findings highlight the multifaceted effects of JAK inhibition on both immune and non‐immune cell populations in periodontitis, emphasizing the complexity of its mechanisms of action.

The limited understanding of JAK signaling in periodontitis underscores the importance of this research. A single study examining the effects of JAK3 inhibition in an experimental model of periodontitis presented conflicting results compared to our findings [13]. This study reported an increase in inflammatory infiltrate density and alveolar bone loss in mice inoculated with *Porphyromonas gingivalis* (*Pg*) and administered an oral JAK inhibitor. Moreover, in vitro tests conducted on immune cells stimulated with *Pg* and treated with the JAK inhibitor demonstrated an elevated production of inflammatory cytokines compared to the control group. It is important to note that the disparities in results might be attributed to the specific experimental model utilized, which encompassed a 43‐day experimental period. Additionally, the method used to induce periodontitis differed from the approach adopted in our research, potentially accounting for the divergent outcomes observed in contrast to our data [13].

The efficacy of different JAK inhibitors (JAK1‐3i vs. JAK3i) in preventing the pathological changes associated with ligature‐induced periodontitis was evaluated, and the results did not demonstrate a clear superiority of either compound. Both inhibitors effectively prevented bone resorption and protected against the inflammatory status of gingival tissue, hallmarks of periodontitis, without causing adverse effects in the animals. Nonetheless, subtle differences were observed: JAK1‐3i was slightly more effective in reducing CD45+ leukocyte infiltration and preserving collagen fibers. This enhanced effect is likely attributable to its broader range of action on inflammatory mediators, since JAK1 and JAK2 are involved in multiple cytokine signaling pathways that modulate both innate and adaptive immunity. In contrast, JAK3 acts more selectively on lymphocyte‐mediated pathways. These distinctions may also be particularly relevant in the preventive phase of disease, as modeled in our study, where broader immunomodulation could be more effective in halting the onset of tissue breakdown. Therefore, the relative efficacy of selective versus broader JAK inhibitors may depend not only on their molecular targets but also on the timing of intervention and the immunological mechanisms predominant at different disease stages.

In the broader context of inflammatory diseases, these results contribute to a growing body of evidence supporting the therapeutic potential of JAK inhibition in conditions such as myelofibrosis [37, 38, 39, 40, 41], rheumatoid arthritis [14, 42, 43, 44, 45, 46] and periodontitis [14, 45]. The common point between these diseases is the direct impact on bone tissue as a result of the inflammatory process. Conventional treatments for such conditions often lead to significant side effects in patients, in addition to a high rate of recurrence after completion of treatment. Faced with this challenging scenario, new therapeutic approaches, such as the use of JAK inhibitors, are being thoroughly explored as promising alternatives. The results obtained so far demonstrate significant improvements in the reduction of specific parameters associated with each disease, contributing to improvement in the quality of life of patients who, even after many years of post‐treatment follow‐up, continue to experience lasting benefits [46].

Despite the promising findings regarding JAK inhibition in our experimental model, it is important to consider potential limitations for its translational application. Systemic administration of JAK inhibitors has been associated with adverse effects such as increased susceptibility to infections, particularly in patients with chronic inflammatory conditions or under long‐term treatment regimens. However, the potential utility of JAK inhibitors in periodontitis may differ substantially from their application in systemic autoimmune diseases. Periodontitis, although chronic, is a localized inflammatory condition that may not require sustained systemic immunosuppression. A plausible clinical application could involve the short‐term adjunctive use of JAK inhibitors during periods of active disease or refractory inflammation, aiming to restore immune balance and promote resolution when conventional therapy alone is insufficient. In this context, strategies that limit systemic exposure, such as local drug delivery directly into periodontal tissues, could enhance safety while preserving therapeutic efficacy. Further investigations should address these aspects to better define the clinical feasibility and scope of JAK inhibition as an adjunctive therapy in periodontal treatment.

Our study has some limitations, including the use of a single inhibitor dose based on previous literature, a single experimental time point (7 days), and the exclusive use of male rats, which may limit the generalizability of the findings. Additionally, due to limited protein yield from gingival tissues, only TNF‐α was assessed by ELISA. Although IL‐6 quantification was initially planned, sample constraints precluded its analysis. Future studies should consider these limitations and explore local delivery methods and models of periodontal repair to better define the potential of JAK inhibition as an adjunctive strategy in periodontitis management.

## 5 Conclusion

Inhibition of the JAK pathway using JAK1‐3 and JAK3 inhibitors significantly modulated immune responses and attenuated tissue degradation in a rat model of experimentally induced periodontitis. These findings enhance our understanding of the molecular and cellular mechanisms underlying JAK signaling in periodontal inflammation and tissue destruction. While the results suggest potential avenues for adjunctive therapeutic strategies, further translational studies are needed to determine the safety and efficacy of targeting the JAK pathway in the clinical setting.

## Author Contributions

M.A.G. and F.R.M.L. contributed substantially to the conception and design of, or acquisition of data or analysis and interpretation of data. A.C.C., K.G.A.G., V.B.C., and I.A.F.M. acquired and analyzed the data. E.P. and F.R.M.L. interpreted the data and participated in drafting the article or revising it critically for important intellectual content. M.R.G.‐S. approved the final version to be published.

## Conflicts of Interest

The authors declare no conflicts of interest.

## Supporting information


**Appendix S1:** jre70042‐sup‐0001‐AppendixS1.zip.

## Data Availability

The data that support the findings of this study are available from the corresponding author upon reasonable request.
