# Supplementary material for: JAK Inhibition Prevents Bone Loss and Reduces Inflammation in Experimental Periodontitis
Source: J Periodontal Res. 2025 Oct 3;60(10):1039–49. doi: 10.1111/jre.70042 (PMC12640214; doi:10.1111/jre.70042)
Supplement: Supplementary file 1 — Appendix S1: jre70042‐sup‐0001‐AppendixS1.zip. [file JRE-60-1039-s001.zip › jre70042-sup-0002-Supinfo.docx]

**JAK inhibition prevents bone loss and reduces periodontal inflammation in an experimental rat model of periodontitis.**

**Supplementary Material:**

**Supplementary Table 1**. Experimental groups (n=40) according to the compound administered and the induction of periodontitis.

| Groups | Periodontitis Induction | Procedure  (Systemic Administration) |
| --- | --- | --- |
| Control | No | Distilled water |
| Experimental Periodontitis (EP) | Yes | Distilled water |
| JAK1-3i | Yes | JAK1-3 Inhibitor (6.2mg/kg) |
| JAK3i | Yes | JAK3 Inhibitor (6.2mg/kg) |

**
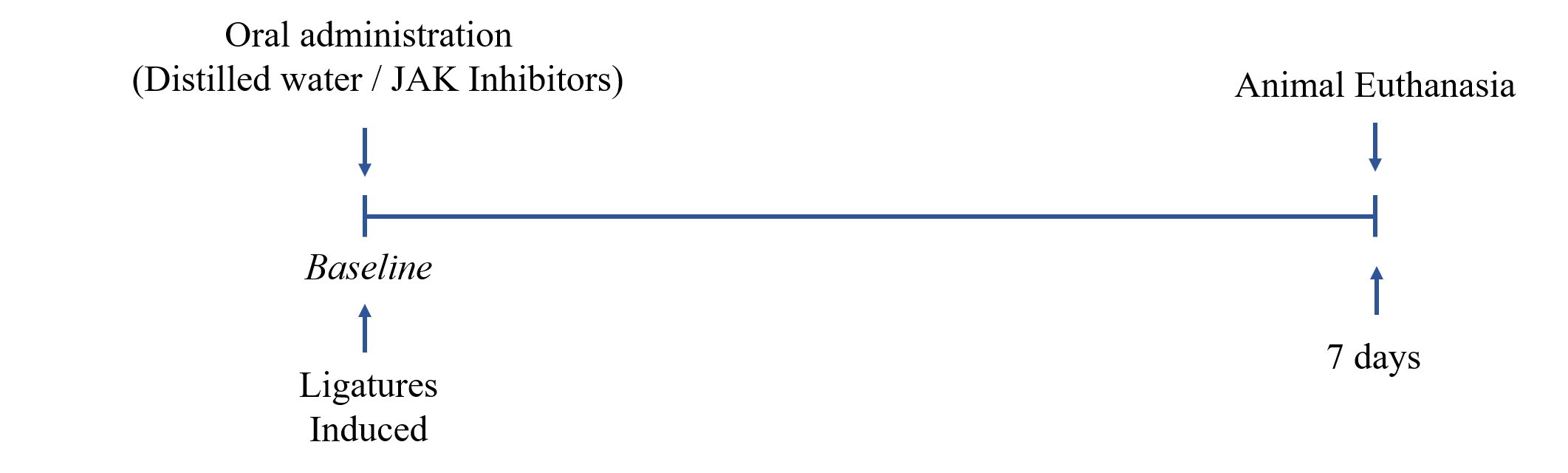
**

**Supplementary figure 1.** Timeline demonstrating the experimental period, administration of distilled water and JAK inhibitors, as well as the euthanasia period.


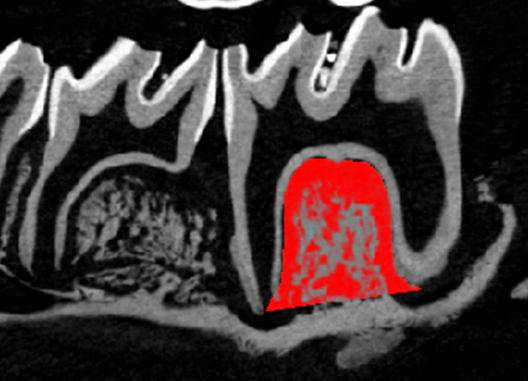


**Supplementary figure 2**. Representative image of the standardized region of interest (ROI), defined in sagittal orientation in the furcation area of the lower first molar, extending from the top of the furcation to the root apices.
