# Supplementary material for: JAK Inhibition Prevents Bone Loss and Reduces Inflammation in Experimental Periodontitis
Source: J Periodontal Res. 2025 Oct 3;60(10):1039–49. doi: 10.1111/jre.70042 (PMC12640214; doi:10.1111/jre.70042)
Supplement: Supplementary file 1 — Appendix S1: jre70042‐sup‐0001‐AppendixS1.zip. [file JRE-60-1039-s001.zip › jre70042-sup-0001-Figures.docx]

**
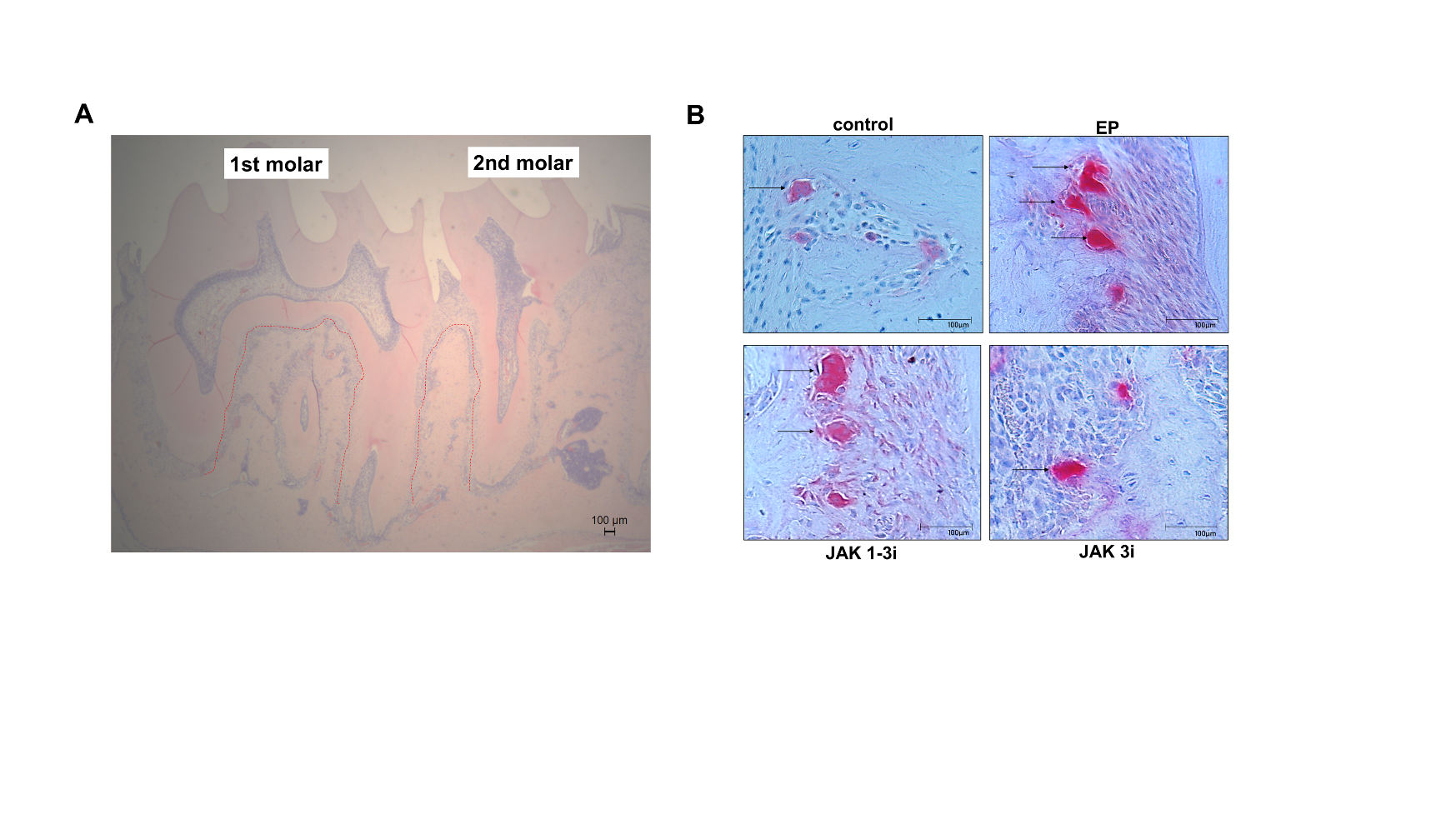
**

**Supplementary Figure 1**: Lower magnification and representative images of TRAP-stained sections used for osteoclast quantification. (A) Lower magnification image showing the standardized anatomical regions used for osteoclast quantification: the interproximal area between the first and second molars and the furcation region of the first molar, as delimited by the red dotted lines. (B) Representative higher magnification images of TRAP staining in each experimental group, highlighting differences in the number of TRAP-positive multinucleated osteoclasts (indicated by black arrows). These images complement the quantitative data presented in Figure 1C.


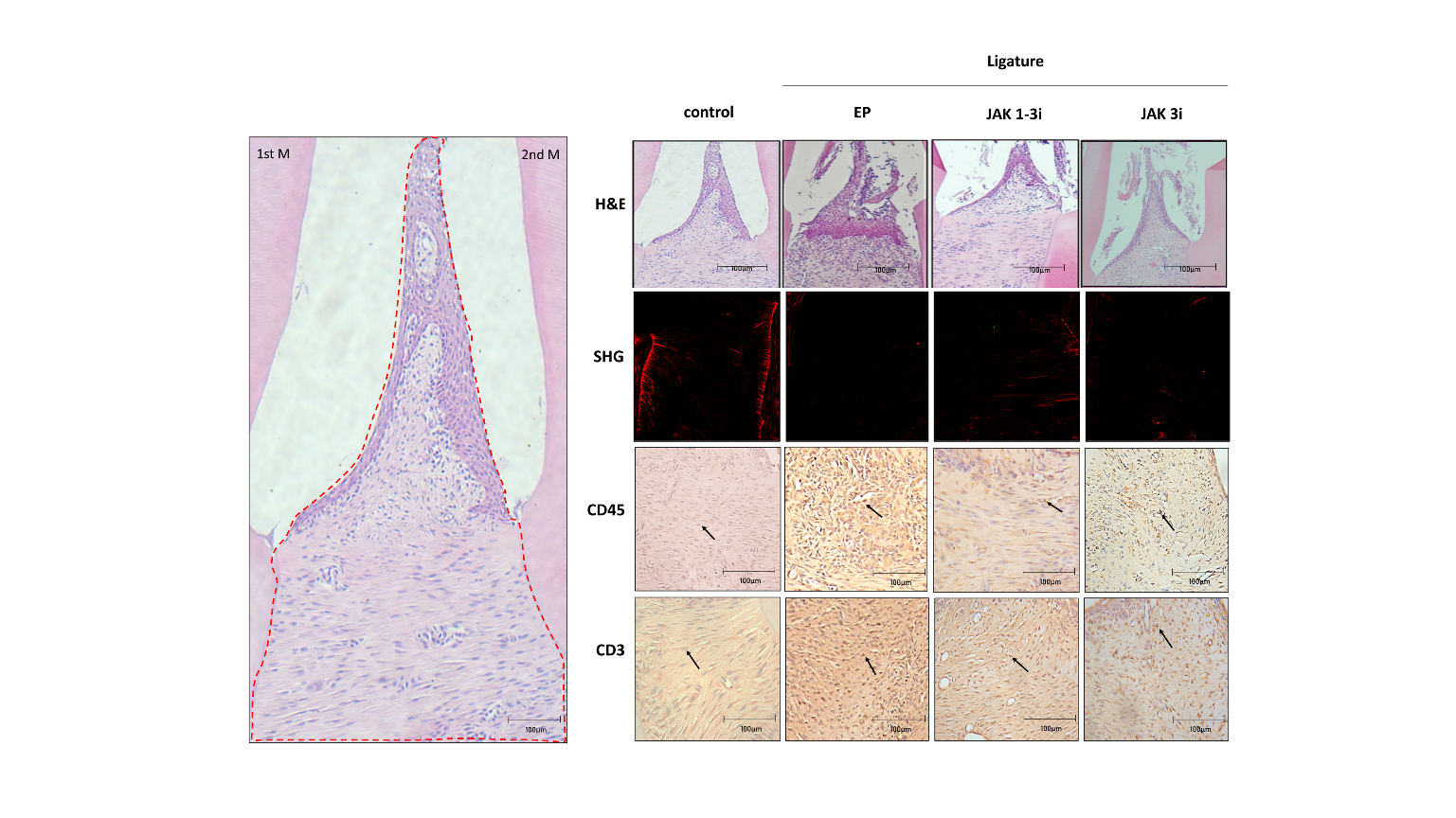


**Supplementary Figure 2:** The H&E-stained image in the left panel illustrates the region of interest (ROI), located in the interproximal area between the mandibular first and second molars, outlined by a red dashed line. This same ROI was consistently used for all histological analyses presented in this figure. Representative histological images used for stereometric analysis, Second Harmonic Generation (SHG) microscopy, and CD45 and CD3 immunohistochemistry are shown (right panel). Arrows indicate CD45⁺ or CD3⁺ cells in the respective immunohistochemistry images. 1st M: Mandibular First Molar; 2nd M: Mandibular Second Molar.


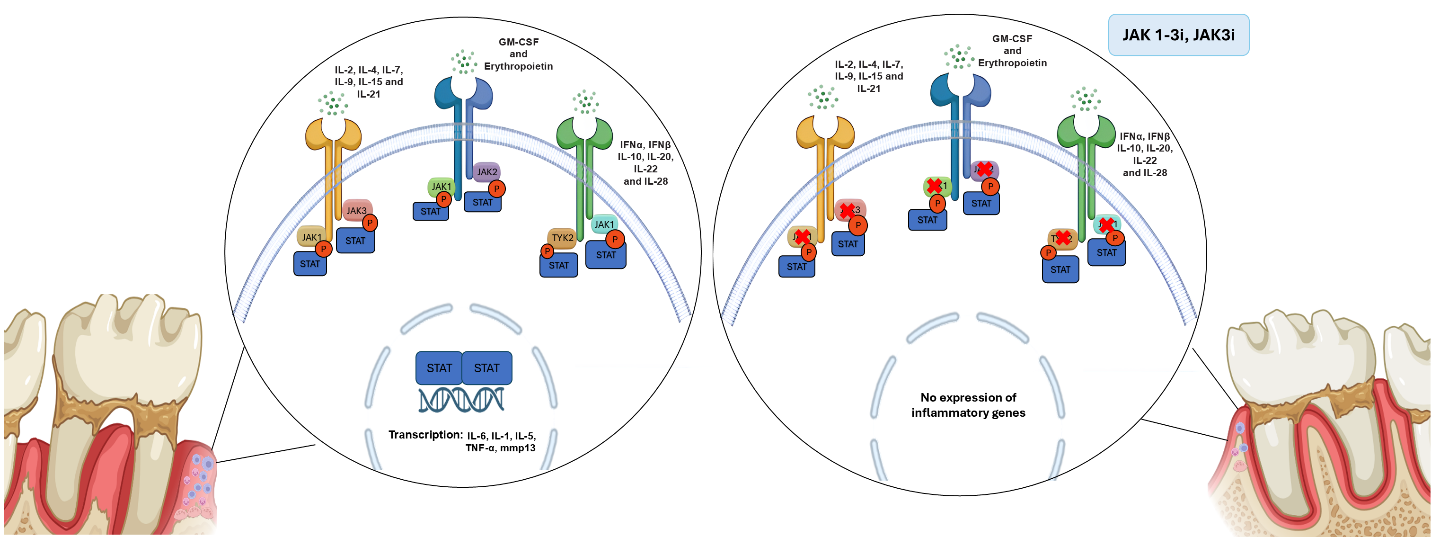


**Supplementary Figure 3:** Schematic summary of the JAK signaling pathway and the main effects observed after JAK inhibition in experimental periodontitis. The illustration highlights how JAK inhibition modulates inflammatory mediators, reduces osteoclastic activity, and preserves tissue integrity, integrating the molecular and cellular mechanisms underlying the protective effects observed in this study.
